# Treadmilling analysis reveals new insights into dynamic FtsZ ring architecture

**DOI:** 10.1371/journal.pbio.2004845

**Published:** 2018-05-18

**Authors:** Diego A. Ramirez-Diaz, Daniela A. García-Soriano, Ana Raso, Jonas Mücksch, Mario Feingold, Germán Rivas, Petra Schwille

**Affiliations:** 1 Department of Cellular and Molecular Biophysics, Max Planck Institute for Biochemistry, Martinsried, Germany; 2 Graduate School for Quantitative Biosciences (QBM), Ludwig-Maximillians-University, Munich, Germany; 3 Centro de Investigaciones Biológicas, Consejo Superior de Investigaciones Científicas (CSIC), Madrid, Spain; 4 Department of Physics, Ben Gurion University, Beer Sheva, Israel; MRC Laboratory of Molecular Biology, United Kingdom of Great Britain and Northern Ireland

## Abstract

FtsZ, the primary protein of the bacterial Z ring guiding cell division, has been recently shown to engage in intriguing treadmilling dynamics along the circumference of the division plane. When coreconstituted *in vitro* with FtsA, one of its natural membrane anchors, on flat supported membranes, these proteins assemble into dynamic chiral vortices compatible with treadmilling of curved polar filaments. Replacing FtsA by a membrane-targeting sequence (mts) to FtsZ, we have discovered conditions for the formation of dynamic rings, showing that the phenomenon is intrinsic to FtsZ. Ring formation is only observed for a narrow range of protein concentrations at the bilayer, which is highly modulated by free Mg^2+^ and depends upon guanosine triphosphate (GTP) hydrolysis. Interestingly, the direction of rotation can be reversed by switching the mts from the C-terminus to the N-terminus of the protein, implying that the filament attachment must have a perpendicular component to both curvature and polarity. Remarkably, this chirality switch concurs with previously shown inward or outward membrane deformations by the respective FtsZ mutants. Our results lead us to suggest an intrinsic helicity of FtsZ filaments with more than one direction of curvature, supporting earlier hypotheses and experimental evidence.

## Author summary

FtsZ is a tubulin homologue and the primary protein of the bacterial Z ring that guides cell division. In vivo, but also in reconstituted systems, FtsZ shows an intriguing treadmilling dynamic along circular tracks of approximately 1 micrometer in diameter. In cells, this treadmilling along the circumference of the division site is suggested to dynamically guide peptidoglycan—and thus new cell wall—synthesis. In vitro, when reconstituted along with its membrane adaptor FtsA on flat supported membranes, FtsZ self-organizes into similarly treadmilling vortices as observed in vivo but with a clear chirality. With the aim of thoroughly investigating these dynamics, revealing the origin of chirality, and potentially relating it to a membrane-transforming ability of FtsZ, we reconstituted different membrane-targeted mutants of FtsZ on flat membranes. In this minimized system, we found that dynamic ring formation is an intrinsic feature of FtsZ without the need of any other protein. However, self-organization into dynamic treadmilling only occurs within a specific protein, cation, and guanosine triphosphate (GTP) concentration range. Our work led us to propose that the observed chirality of FtsZ treadmilling may be explained by an inherent helical character of the filaments with more than one direction of curvature.

## Introduction

For the fundamental task of cell and organelle division, life has evolved various strategies, many of which are based on ringlike contractile structures assembling from within a compartment to induce binary fission. The exact mechanisms of contraction of these rings are, however, poorly understood due to the plethora of different molecules involved, concealing presumably simple fundamental motifs. The bacterial FtsZ ring is a good example of such a structure. The FtsZ protein, a self-assembling GTPase present in the cytoplasm of most bacteria, is a tubulin homologue and the main component of the *E*. *coli* divisome, the molecular machinery driving cytokinesis [1,2]. It interacts with additional anchor proteins to form a dynamic ring at the cytoplasmic membrane, which acts as a scaffold to recruit the remaining regulating elements of the divisome and cell wall–building machinery [3]. Intriguingly, FtsZ filaments were recently found to treadmill circumferentially around the division plane, guiding cell wall synthesis [4,5]. In spite of this compositional complexity, the primary role of FtsZ in the contractile ring of many organisms is unquestioned. However, based on the structural analysis of the protein and its assemblies, so far, no decisive model has surfaced for how exactly structural changes within monomers and filaments could be transmitted into large-scale contractile forces [6]. Guanosine triphosphate (GTP)-dependent structural changes of FtsZ monomers and membrane-attached filaments toward greater curvatures have been proposed, but evidence has been lacking for how a continuously shrinking membrane orifice could be engineered from them. This is mainly due to the fact that although the closure dynamics of Z rings could be observed in vivo [7,8], there is yet no direct proof that purified Z rings may actively proceed to closure through all stages of increasing curvature [3,9].

For membrane attachment, FtsZ requires either FtsA or ZipA. Together they form the so-called proto-ring, the first molecular assembly of the divisome [2,10]. FtsA is an amphitropic protein that associates to the membrane by an ATP-linked process mediated by a short amphipathic helix [11]. The bitopic membrane protein ZipA contains a short N-terminal region facing toward the periplasmic space, a transmembrane region, and a C-terminal FtsZ-interacting domain connected by a flexible linker region [12]. FtsZ binds the proto-ring tethering elements through its C-terminal end, which is also the interaction region for FtsZ-regulating proteins such as MinC, inhibiting FtsZ polymerization and hence FtsZ ring formation at undesired locations. Thus, the C-terminal region of FtsZ acts as a central hub, integrating signals that modulate divisome assembly in *E*. *coli* [2]. The membrane anchor proteins can be bypassed by an FtsZ chimera—FtsZ–yellow fluorescent protein (YFP)–membrane-targeting sequence (mts)—through replacing the FtsZ central hub with a YFP and an amphipathic helix to provide autonomous membrane attachment. This mutant was found to become internalized and accumulated in narrow regions of tubular liposomes, forming ringlike structures. Polymerization of this chimeric FtsZ protein at the external face of liposomes has also shown to induce inward or outward deformations, depending on the location of the mts (C-terminus or N-terminus) [13,14].

Recently, the coreconstitution of FtsZ and FtsA on supported bilayers has revealed that FtsA promotes the self-organization of FtsZ fibers into dynamic patterns, giving rise to coordinated streams and swirling rings with preferential directions, due to treadmilling dynamics [15]. In contrast, this dynamic behavior was not observed when FtsZ was tethered to the membrane through ZipA or when the membrane-targeted FtsZ variant was used. Interestingly, FtsZ–FtsA dynamic vortices showed no apparent change in size and curvature, suggesting that the functional role of the energy-consuming circumferential movement of FtsZ in a ring of apparently conserved radius was a secondary one: i.e., the sequential spatial targeting of downstream enzymes for cell wall assembly. There was further evidence by recent in vivo studies demonstrating the circumferential treadmilling dynamics of FtsZ in dividing cells and directly connecting it to spatial targeting of peptidoglycan synthesis [4,5]. However, in spite of a wave of new papers targeting the exact role of FtsZ in ring architecture, the current models of how exactly the Z ring is constricting over time, and how the large accessible range of FtsZ filament curvatures may be exploited in this process, are still disappointingly vague.

To study the intrinsic role of FtsZ in the formation of dynamic patterns, we thus revisited the in vitro reconstitution of the membrane-targeted chimeric FtsZ variant, FtsZ-YFP-mts, to flat supported membranes. We further addressed the question whether the pronounced spatial dynamics in FtsZ vortices on the membrane could constitute a direct, rather than indirect, spatial cue in the constriction process of the Z ring. To this end, we compared FtsZ mutants that showed different deformation phenotypes when targeted to free-standing membranes.

Our results can be summarized as follows:

1. In contrast to what was proposed by Loose and Mitchison, the membrane adaptor FtsA is not required for the emergent spatial self-organization dynamics of FtsZ. Its role can be fully accounted for by an mts to FtsZ.
2. A narrow range of protein concentrations on the membrane, modulated by free Mg^2+^ and dependent on GTP hydrolysis, supports the formation of dynamic FtsZ swirls.
3. Treadmilling results from a directional growth of curved and polar filaments from nucleation points at the membrane. The preferential addition of GTP subunits to the leading edge establishes a GTP–guanosine diphosphate (GDP) gradient along membrane-attached filaments, in which the “older” GDP-rich end is more likely to become destabilized.
4. There is a striking relationship between the vortex chirality of treadmilling and membrane attachment and deformation. FtsZ with C-terminal mts bending the membrane toward the attachment interface treadmills clockwise, while the N-terminal attachment mutant bending the membrane away from the attachment interface treadmills counterclockwise.

## Results

### Membrane-targeted FtsZ self-organizes into dynamic rotating vortices

**Fig 1:**
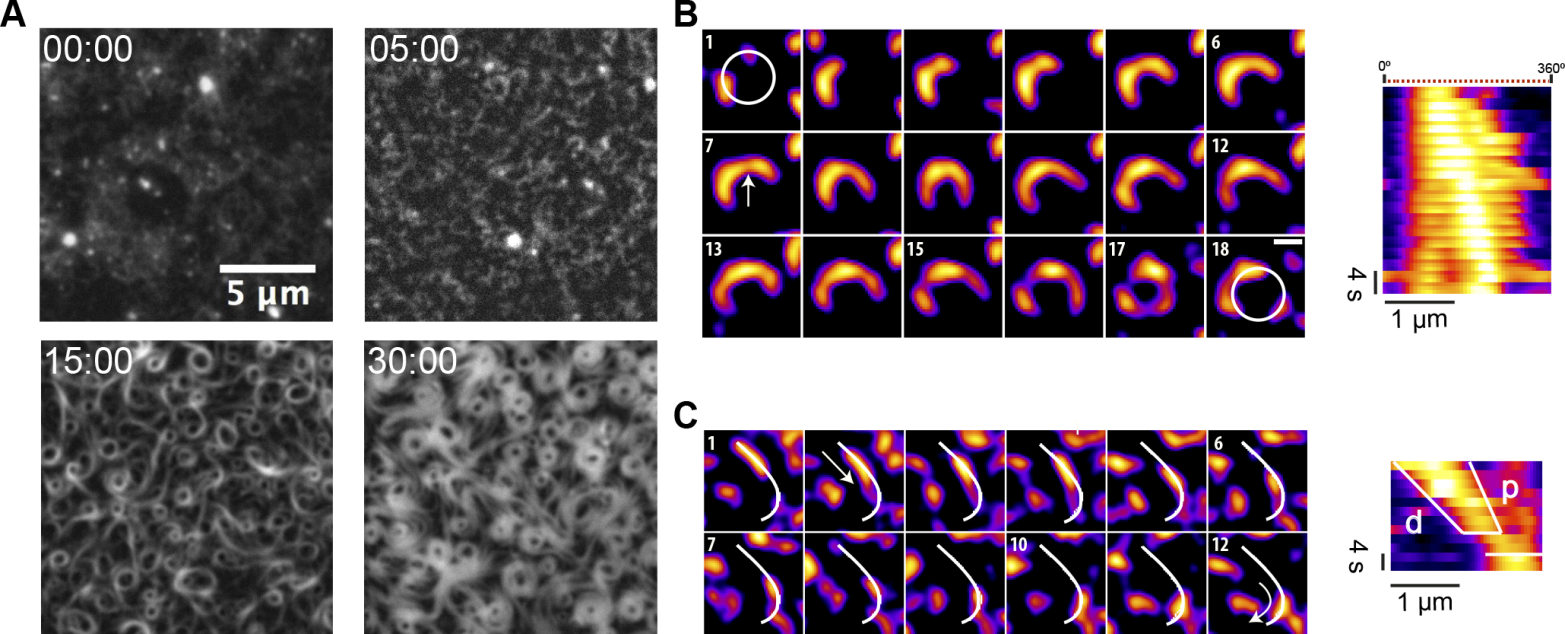
Nucleation and growth of FtsZ filaments into rings on SLBs. (A) Representative snapshots from a time-lapse experiment displaying different stages of ring formation. Images were taken every 10 s using TIRF illumination (YFP channel). Frames correspond to the times (min) indicated after the addition of GTP and Mg^2+^. (B) Polar clockwise growth of a single FtsZ from a nucleation point. Growth seems to occur stepwise and depend on the accessibility of small filaments nearby (panel 1 and 2). Lower local protein density (white arrow) correlates with a higher flexibility of the polymer (panels 7–12). Breakage occurs primarily in trailing regions (15). After about 3 min, a primitive ring made of 3 distinct short filaments is exhibited (17). (C) Directional filament gliding via treadmilling. Fragmentation or depolymerization destabilizes the trailing (“older”) edge as shown in the kymograph (d-labeled white line). Images in (B) and (C) were taken every 2 s, and the scale bar represents 500 nm. Further details are under “Results.” Movies reconstructed from the whole collection of images can be found in supporting information S2 and S3 Movies. GTP, guanosine triphosphate; SLB, supported lipid bilayer; TIRF, total internal reflection fluorescence; YFP, yellow fluorescent protein.

The protein chimera FtsZ-YFP-mts (0.5 μM) in its GDP-bound form (corresponding to a nonassembled state, according to sedimentation velocity, S1 Fig) did not form visible structures on a supported lipid membrane, as revealed by a total internal reflection fluorescence microscope (TIRFM). We have found that FtsZ-YFP-mts under assembly-promoting conditions (4 mM GTP, 5 mM Mg^2+^) formed filaments on supported lipid bilayers (SLBs), which self-organize with time into dynamic ringlike structures (S1 Movie). The assembly of the dynamic rings is a time-dependent phenomenon. After several minutes of GTP addition—during which highly dynamic short filaments were observed to attach, detach, and diffuse on the surface—longer curved filaments appeared to grow directionally (Fig 1A panel 5:00). At this stage, intrinsic motion drives filament–filament interactions to create small and dim closed circular structures (Fig 1A panel 15:00). These structures tend to be highly unstable: Closed filaments were able to open, to fuse with adjacent filaments, or to close back. At later times, closed circular strands turned into thicker ringlike structures (Fig 1A panel 30:00).

To understand the formation of circular structures and how they evolve into stable and thicker rings, we resolved and tracked individual filaments (consisting of several protofilaments) before stable ring formation. Here, we identified nucleation sites where clockwise chiral growth leads to the formation of circular structures (Fig 1B, S2 Movie). Despite the fact that growth seems to be a discontinuous phenomenon limited by the accessibility of soluble protein, we can estimate a mean growth rate (slope p in the kymograph) which was found to be around 60 nm/s. Strikingly, these images also showed filament flexibility (panels 7–12) and breakage (panels 15–18) resulting in the formation of short free fragments. Such fragments were found to glide and “explore” the surface via treadmilling (Fig 1C, S3 Movie), indicating that this process fuels filament–filament interactions and therefore ulterior formation of closed circular structures. The kymograph in Fig 1C showed a representative example of one filament growing in the leading edge (p-line) and shrinking in the trailing edge (d-line). A coarse estimation of the velocity of displacement of this filament was about 55 nm/s.

### Surface FtsZ concentration—linked to Mg—critically modulates the emergence of dynamic chiral ringlike structures

We further investigated the impact of protein concentration on the stability and dynamics of FtsZ vortex formation in the presence of GTP (4 mM) and Mg^2+^ (5 mM). Below 0.2 μM, no FtsZ filaments could be detected (S2 Fig). Interestingly, increasing the protein concentration to around 1.0 μM resulted in the formation of abundant three-dimensional polymer networks on the membrane, and no dynamic FtsZ rings were observed (S2 Fig, [15]). These results showed that the self-organization behavior of membrane-targeted FtsZ polymers was critically dependent on total protein concentration.

**Fig 2:**
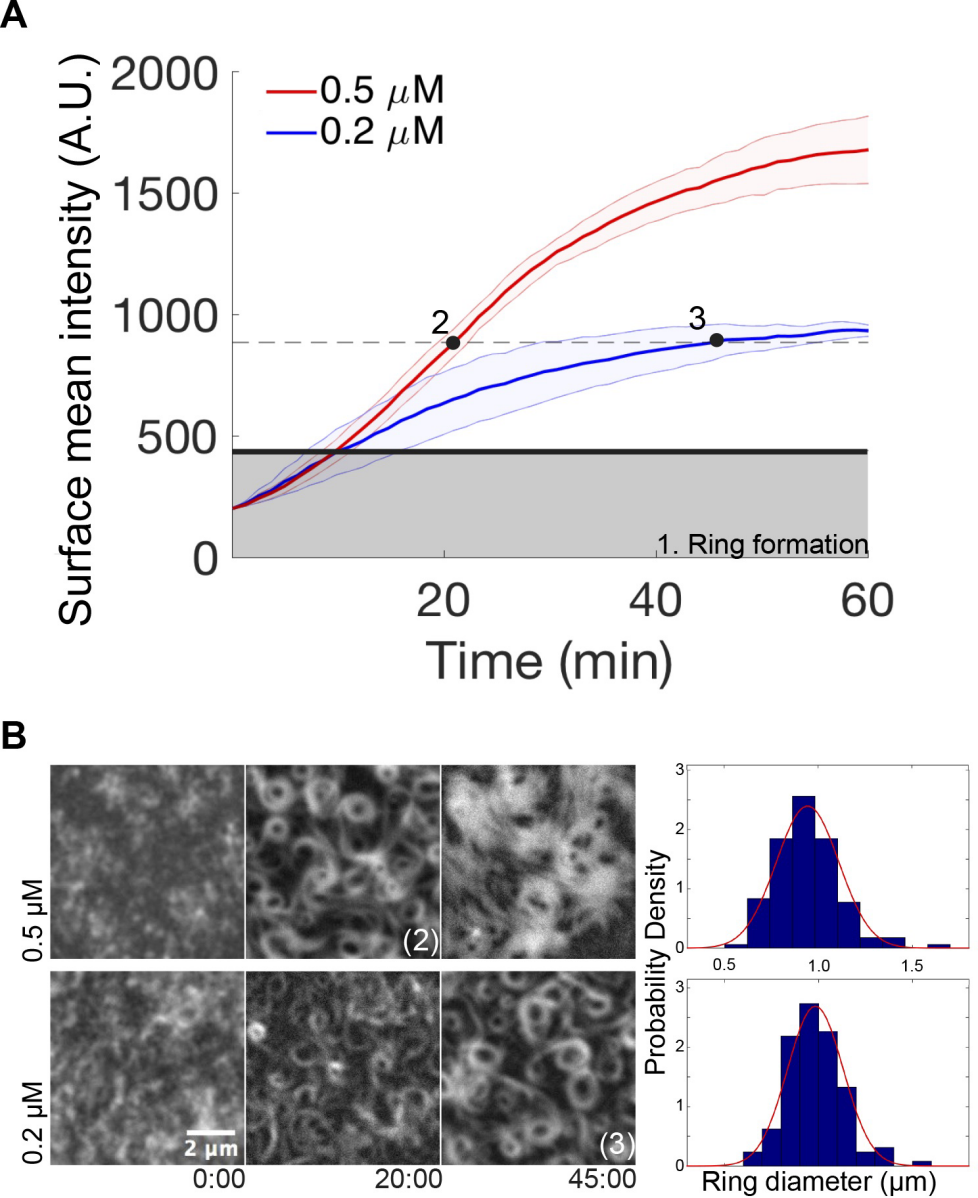
Dependence of FtsZ-YFP-mts vortex formation on protein surface concentration. (A) Time dependence of the average fluorescence intensity of FtsZ-YFP-mts on the bilayer upon 4 mM GTP and 5 mM Mg^2+^ addition, as measured by TIRFM, at 0.2 (blue line) and 0.5 (red line) μM protein concentration. The gray area marks the intensity when first closed rings are observed, which is approximately the same for both protein concentrations. After closed rings have formed, the further accumulation of protein at the surface is strongly concentration dependent. The dashed line represents the phase in which clearly discernible, locally stable dynamic vortices are observed. While at 0.2 μM, the system reaches this regime after an elapsed time of 45 min (time point 3); at 0.5 μM, it only takes approximately 20 min (time point 2). (B) Representative images of the experiment shown in panel (A). Frames were taken at elapsed times in minutes. Right: Ring size distributions at time points 2 and 3, indicated in panel (A), with average diameters of 0.94 +/− 0.16 μm, *N* = 140, and 0.98 +/− 0.14 μm, *N* = 128, respectively. Size distributions of rings are similar since both correspond to the same protein surface density (approximately 880 A.U.) Further details are under “Results.” A.U., arbitrary units; GTP, guanosine triphosphate; mts, membrane-targeting sequence; TIRFM, total internal reflection fluorescence microscope; YFP, yellow fluorescent protein.

Next, we compared the kinetics of protein binding to the membrane at 0.2 μM and 0.5 μM of protein (Fig 2A), under conditions previously used to detect the swirling rings (see Fig 1). Upon the addition of GTP (4 mM) and Mg^2+^ (5 mM), a similar membrane adsorption rate (Fig 2A) and the parallel appearance of short and highly dynamic filaments (Fig 2B) were initially found for the two protein concentrations. Remarkably, the transition from short filaments to rudimentary circular structures (gray area in Fig 2A) also occurred at similar times in both cases. After a lag time of around 10 min, the adsorption rate was found to be significantly slower at 0.2 μM than at 0.5 μM, suggesting that the kinetics of ring stabilization and widening of the structures was concentration dependent (Fig 2A). These differences, found at elapsed times greater than 10 min, also correlated with the fact that the morphology of the rings observed at a protein concentration of 0.2 μM after 45 min incubation (Fig 2B, bottom right panel) was similar to the ones obtained after a lag time of 20 min when 0.5 μM protein was used (Fig 2B, upper mid panel). The morphological similarity found at these two time points (denoted as 2 and 3 in Fig 2A) occurred at a similar protein coverage of the membrane, suggesting that protein surface density, rather than bulk concentration, is the key parameter determining the nature of the network that assembles on the membrane. The correlation between the morphologies at time points 2 and 3 of Fig 2A was further established by determining the average diameter of the formed rings to be about 1 μm at both protein concentrations (Fig 2B). This suggests that although the adsorption rates were different at these time points, the proteins condensed into similar structures.

**Fig 3:**
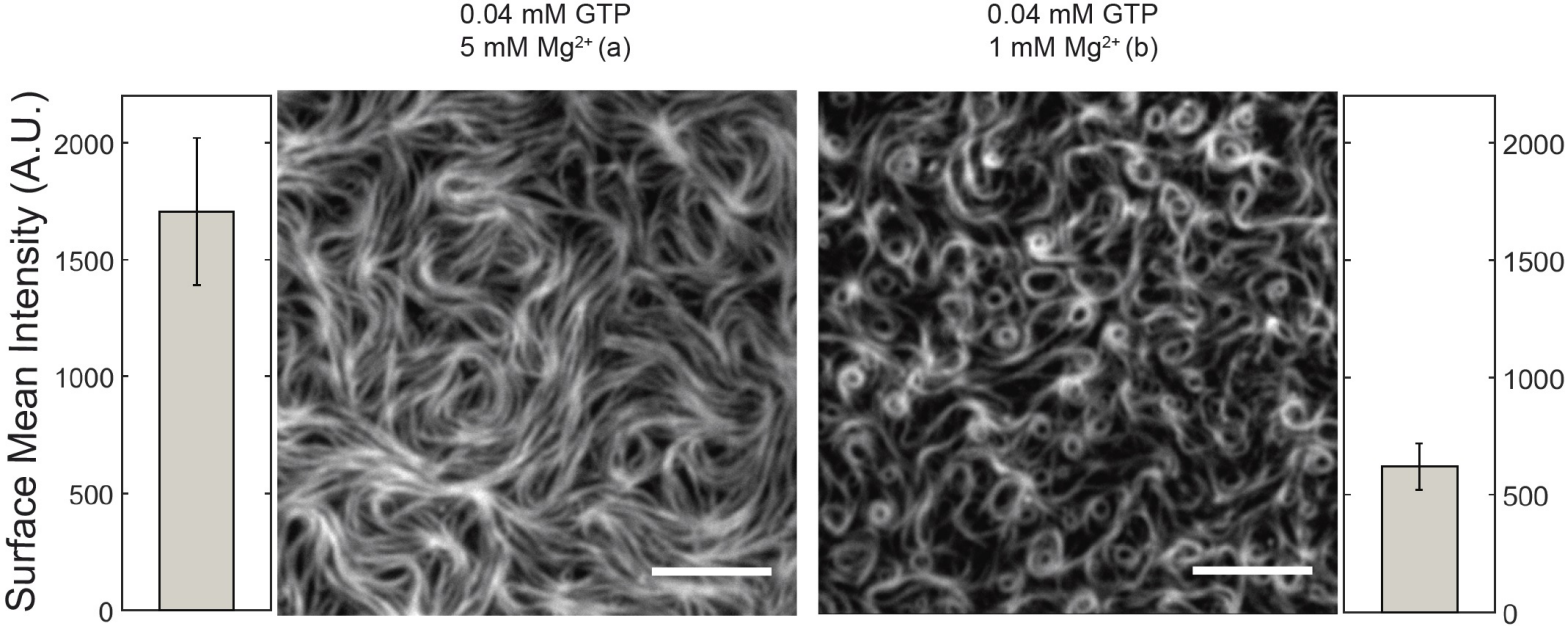
Free Mg^2+^ regulates protein surface concentration and thus self-organization of membrane-targeted GTP-FtsZ. Representative snapshots showing TIRFM images of FtsZ-YFP-mts (0.2 μM) polymers on the bilayer taken 2–3 min after the addition of 0.04 mM GTP in the presence of 1 mM and 5 mM free Mg^2+^ concentrations, respectively. Next to each image, the mean fluorescence intensity, proportional to the FtsZ-YFP-mts density on the membrane, is shown (average of 3 experiments). The protein network observed at 5 mM free Mg^2+^ correlates with a high FtsZ-YFP-mts density regime, at least 3-fold larger (approximately 1,500 A.U.) than required for ring formation (approximately 500 A.U.) The scale bar represents 5 μM. A.U., arbitrary units; GTP, guanosine triphosphate; mts, membrane-targeting sequence; TIRFM, total internal reflection fluorescence microscope; YFP, yellow fluorescent protein.

Then, we monitored the impact of GTP concentration (0.04, 0.4, and 4 mM) on the formation of swirling vortices at fixed protein (0.2 μM) and Mg^2+^ (5 mM) concentration. Surprisingly, at the lowest GTP assayed (0.04 mM), a highly ordered mesh of static filaments was found at *t* = 0. These filaments retained a certain degree of curvature and behaved as a nematic phase that entirely covered the membrane area (Fig 3, left panel). A similar behavior was also observed at intermediate GTP concentration (0.4 mM) (S3 Fig). Notably, the surface mean intensity is 3-fold (approximately 1,500) increased, compared to the minimal density to form rings (Fig 2), suggesting that the parallel arrangement of filaments correlates with a high-density regime of protein. Furthermore, aligned filaments showed no significant change after 10 min, in contrast to dynamic rings (S3 Fig).

It is known that Mg^2+^ favors self-association and assembly of FtsZ both in solution and at membranes [16,17]. Therefore, one possibility could be that the free Mg^2+^ controls the surface protein density, rather than the total GTP concentration, since GTP is known to bind Mg^2+^ with affinity in the millimolar range [18]. To examine this alternative, we repeated the self-organization assays at 0.04 mM GTP in the presence of 1 mM free Mg^2+^, which resulted in the formation of chiral vortices (Fig 3, right panel). Interestingly, the emergence of chiral vortices was found to correlate with a significantly lower mean surface protein density than the one measured at 0.04 mM GTP and 5 mM Mg^2+^ that resulted in the dense packing of static polymers (Fig 3, left panel). These findings show that free Mg^2+^ controls the concentration of GTP FtsZ-YFP-mts polymers at the membrane and then the self-assembly of the FtsZ filaments in the membrane.

### Directionality of vortices and destabilization of the trailing edge

**Fig 4:**
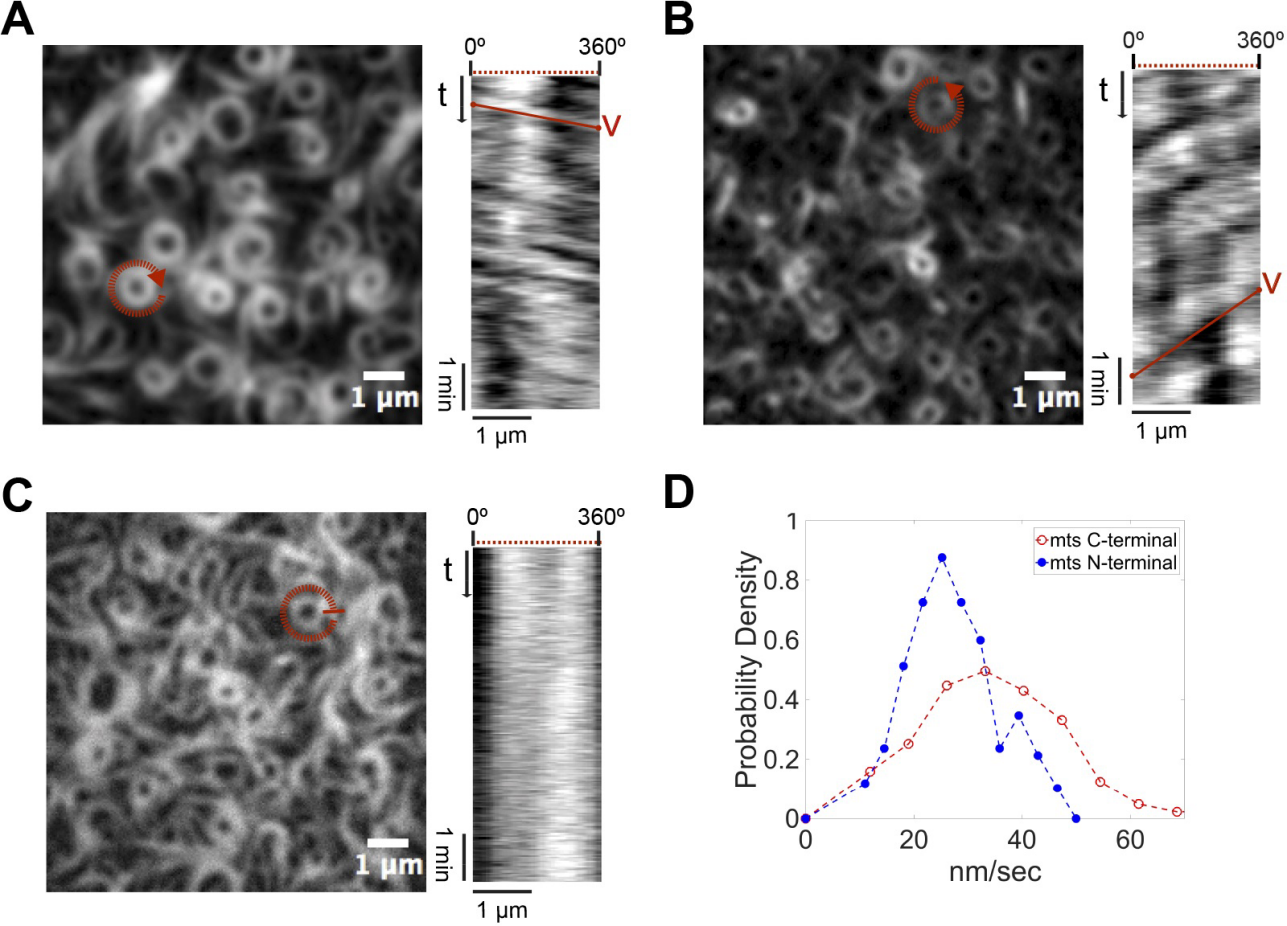
Steady-state treadmilling and chirality of FtsZ vortices: Dependence on GTPase activity and location of the mts. (A-C) Left panels: Representative snapshots of the rings formed upon addition of GTP (4 mM) and Mg^2+^ (5 mM) by (A) FtsZ-YFP-mts, whose mts is located at the C-terminus of FtsZ; (B) mts-H-FtsZ-YFP, with mts located at the N-terminus of FtsZ; and (C) FtsZ*[T108A]-YFP-mts, a variant of FtsZ-mts with diminished GTPase activity. Right panels: Kymograph analysis showing (A) a positive slope that corresponds to the apparent clockwise rotation time of the selected ring (red circle); (B) a negative slope that corresponds to an apparent counterclockwise rotation, indicating that the position of the mts determines the chirality of the apparent rotation; (C) no apparent slope corresponding to static rings, suggesting that the apparent rotation in (A) and (B) is mediated by GTP hydrolysis. (D) Velocity distributions for FtsZ-YFP-mts (red) and mts-H-FtsZ-YFP (blue) with mean rotational speed values of 34 nm/s and 25 nm/s, respectively. Further details under Materials and methods and Results. GTP, guanosine triphosphate; mts, membrane-targeting sequence; YFP, yellow fluorescent protein.

After formation, single rings reach a quasi-steady state as rotating vortices, meaning that the light intensity along their perimeter shows a nearly periodic time dependence. These rotating structures formed by the membrane-targeted FtsZ-YFP-mts (mts C-terminal) consistently showed a chiral clockwise rotation (Fig 4A and S4 Movie). The directional ring dynamics were confirmed by the positive slope of kymographs generated along the ring circumference. Quantifying the slope of the kymograph (see Materials and methods and S4 Fig) of *N* = 60 rings, we calculated the velocity distribution with a mean velocity of 34 nm/s or 3.9° sec^−1^ for rings of about 500 nm radius (Fig 4D). Interestingly, the rotational velocities measured here are in good agreement with those reported in vivo (30 nm/s), in spite of the significantly reduced complexity of the reconstituted system [4,5].

We next sought to understand whether there was any relationship between the structural features of the protein and the obviously chiral dynamics of our FtsZ mutants. Hence, we made use of a previously established chimera variant that was shown to have opposite effects on deformable membranes [14,19]. In the presence of GTP, FtsZ-YFP-mts is able to induce inward (concave) deformations on lipid vesicles. Strikingly, when the mts sequence is switched to the N-terminus, an outward (convex) deformation is observed [14]. To study the role of the position of the mts in our dynamic vortices, we carried out similar self-organization assays using an FtsZ chimera in which the membrane attachment was located at the opposite—the N-terminal—end (mts-H-FtsZ-YFP). Upon addition of GTP and Mg^2+^, defined dynamic rings were observed (Fig 4B). Strikingly, now the FtsZ swirls appeared to rotate counterclockwise (S4 Movie), a feature that was confirmed by the negative slope of the kymographs (Fig 4B). As before, we measured the slope of kymographs for *N* = 50 different rings to calculate the velocity distribution with a mean of about 25 nm/s or 2.8° sec^−1^ for a ring of 500 nm radius. This velocity is slower than that of the FtsZ-YFP-mts vortices (34 nm/s) (Fig 4D). These observations show that the positioning of the mts determines the direction of polymerization, as it does for membrane binding and transformation. The fact that the N-terminal mts mutant—without a protein spacer between the FtsZ and the membrane attachment—results in the same qualitative dynamic behavior, although being inverted in chirality, also refutes potential speculations that YFP may take over a necessary (sterical) role of FtsA [15].

To further investigate how exactly GTP hydrolysis influences the formation of collective streams, we carried out similar self-organization assays using a variant of the FtsZ chimera with no GTPase activity (S5 Fig), in which the Threonine at position 108 was replaced by an Alanine (FtsZ*[T108A]-YFP-mts). Well-defined rings similar in size to the ones found with FtsZ-YFP-mts could be observed upon the addition of GTP and Mg^2+^ (Fig 4C). Interestingly, these rings did not seem to treadmill and rotate (S4 Movie), as evidenced by the lack of clear patterns in the kymographs generated to track polymer dynamics (Fig 4C). Interestingly, FtsZ*[T108A]-YFP-mts rings grow from nucleation points in a less dynamic manner compared to FtsZ-YFP-mts (S5 Movie). From these results, we conclude that GTPase activity is not required for the formation but for the quasi-steady-state rotational dynamics of the ring patterns, suggesting that GTPase activity particularly promotes filament destabilization in the trailing edge.

**Fig 5:**
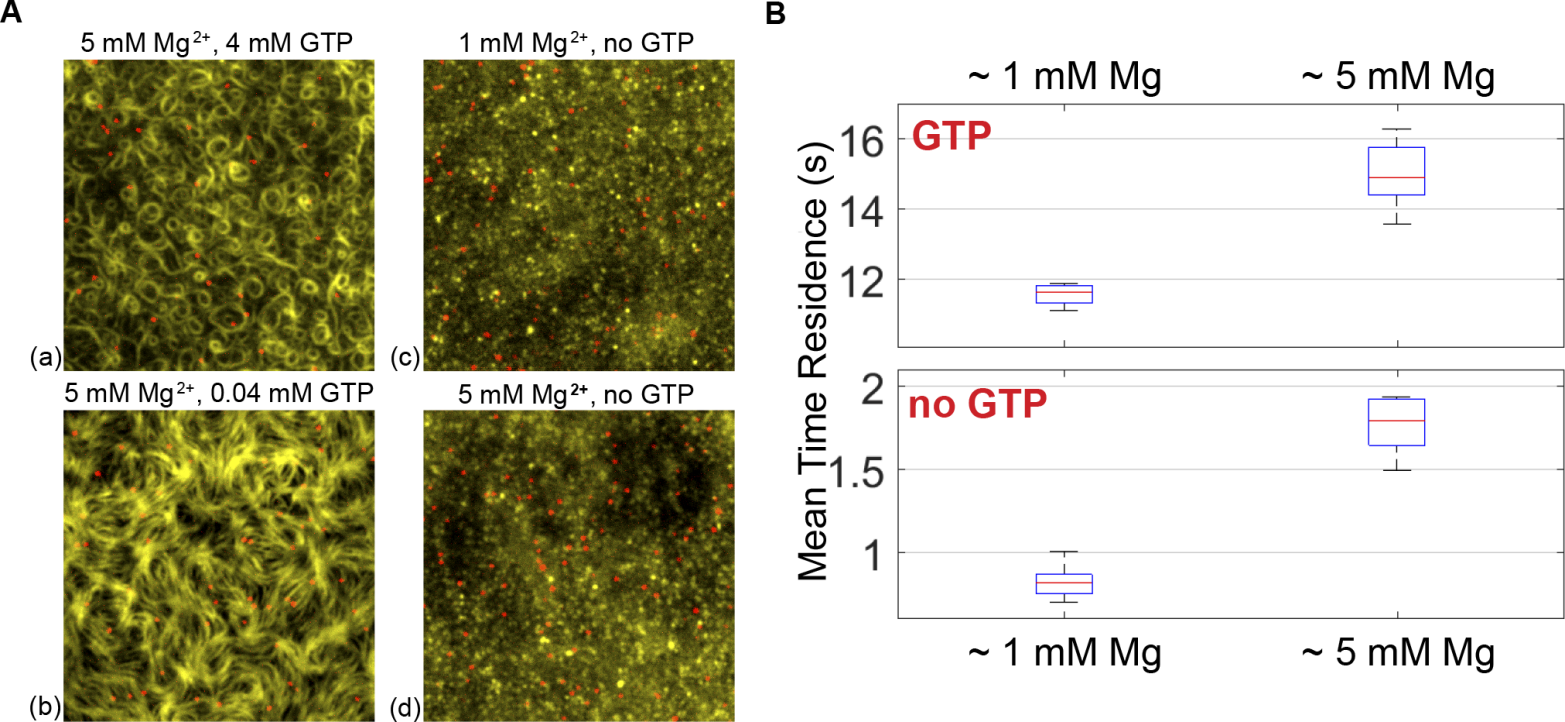
Residence times of single membrane-targeted FtsZ molecules at the bilayer, dependent on nucleotide and free Mg^2+^. (A) Overlaid images of FtsZ-YFP-mts structures (yellow channel) incubated with GFP-Booster-Atto647N (nanobody) (single molecules: red channel) in the presence of GTP (a, b), GDP (c, d), and indicated free Mg^2+^ concentration. The protein concentration in all cases was around 0.2 μM. (B) Mean residence times of FtsZ-YFP-mts were calculated using an exponential fit of the cumulative residence time distribution. Mean residence times were measured for different GTP and Mg^2+^ conditions. Further details under Materials and methods and Results. GDP, guanosine diphosphate; GFP, green fluorescent protein; GTP, guanosine triphosphate; mts, membrane-targeting sequence; YFP, yellow fluorescent protein.

Treadmilling can be explained by an imbalance between growth and shrinkage at the two opposite ends of the polar filament. Since treadmilling is obviously GTP-turnover dependent, and the growth into ringlike structures by capturing preformed diffusing filaments is not, the critical requirement for treadmilling seems to be the destabilization and shrinkage at the trailing edge. In order to directly visualize the destabilization dependent on nucleotide state, we developed a single molecule assay using FtsZ-YFP-mts incubated with fluorescently labeled nanobodies (green fluorescent protein [GFP]-Booster-Atto647N, see Materials and methods) (Fig 5A) to investigate the protein turnover at the membrane, implying that faster disassembly suggests higher destabilization. By measuring the probability of protein detachment as a function of time (S6 Fig), we could calculate the mean residence time of single FtsZ subunits within the filaments on the membrane. Using this analysis, we found that the mean residence time of FtsZ-YFP-mts in fragments forming dynamic rings was $t_{r}=11.5s$ (Fig 5B), in good agreement with previous fluorescence recovery after photobleaching (FRAP) studies with native FtsZ [20,21]. This residence time turns out to be significantly faster than for the GTP hydrolysis–deficient mutant FtsZ*[T108A]-YFP-mts (S6 Fig, similar to the photobleaching time scale contribution approximately 32 s). By considering the rotational speed as measured in Fig 4D and this residence time, we reason that rings are assembled by multiple filaments that treadmill in a synchronized manner, with a mean length of $\langle l\rangle =\underset{-}{v}*t_{res}=390\text{nm}$ (78 monomers). In comparison, in vitro assembly of native FtsZ showed shorter filaments that were, on average, 120 to 200 nm long (30–50 monomers) [21,22]. In addition, we measured the residence time without GTP (GDP form) with 1 mM free Mg^2+^. Assuming that the residence time of a polymer of n-monomers scales to the power of n $\left(t_{r}^{pol},\sim ,{\left(t_{r}^{mon}\right)}^{n}\right)$, one can estimate that the residence time associated to one monomer is approximately 1 s, which agrees with our results (approximately 0.8 s) in GDP form at 1 mM free Mg^2+^.

Our single molecule experiments also allowed us to directly elucidate the impact of free Mg^2+^ and, in particular, its obvious role in the formation of the high-density FtsZ mesh. It was found that the protein release from the membrane upon GTP addition was slower at 5 mM ($t_{r}\;=\;15.1\;s$) than at 1 mM free Mg^2+^ (Fig 5B). These findings represent compelling evidence that the formation of a high-density mesh of filaments is linked to the slow detachment of protein, at least when compared to the dynamic rings at lower free Mg^2+^ (1 mM). Also, the residence time of FtsZ in GDP form at 5 mM free Mg^2+^ was increased ($t_{r}\;=\;1.72\;s$) compared to 1 mM. This general increase in the residence time implies that lateral interactions (bundling), favored by free Mg^2+^, promote larger and more crosslinked polymeric species with higher membrane affinity and less susceptibility to destabilization.

## Discussion

In our minimalistic *in vitro* reconstitution study, we found that polymers of an artificially membrane-targeted variant of FtsZ autonomously and without the presence of FtsA self-organize on a supported bilayer upon addition of GTP and Mg^2+^ to form chiral ringlike dynamic patterns (Fig 1), displaying a clockwise or counterclockwise protein movement, dependent on whether the membrane attachment was enforced through the C-terminal or N-terminal end of the protein, respectively (Fig 4A and 4B). The mts in both cases was taken from MinD, one of the elements of the site-selection MinCDE complex, which allows FtsZ to be peripherally attached to the membrane. We thus showed that the ability of FtsZ to create dynamic patterns is an intrinsic property (Fig 1A) rather than a specific interaction with a specific protein anchor. Instead, the formation of dynamic FtsZ ring structures in vitro is highly linked to (i) the surface protein density and (ii) GTPase activity, destabilizing surface-bound filaments and thus being key for treadmilling [5].

**Fig 6:**
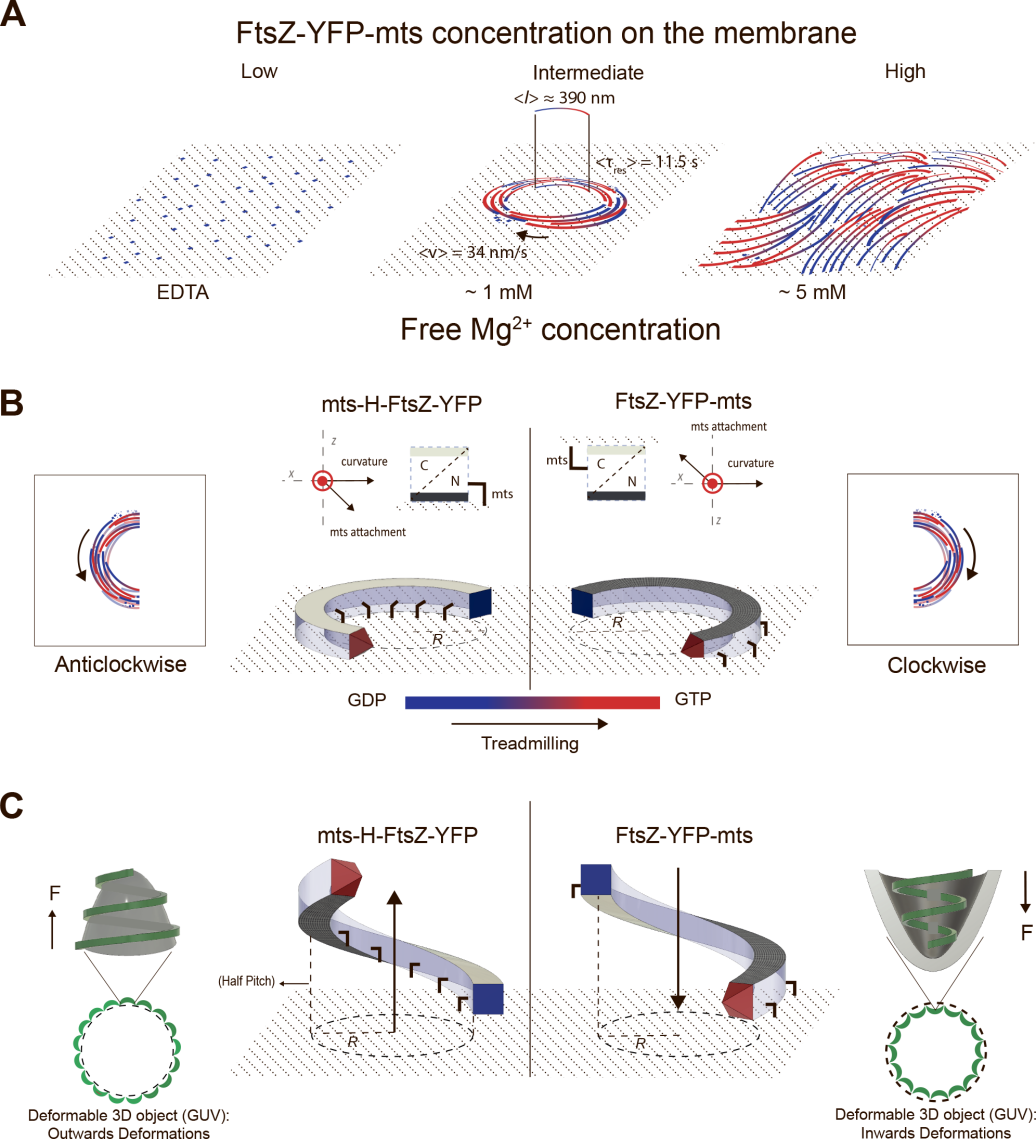
(A) FtsZ-YFP-mts ring formation is dependent on GTP and protein surface concentration. At low protein concentration and no GTP and Mg2+, FtsZ transiently binds to the membrane without forming visible structures. With GTP, dynamic chiral rings are formed as a function of time. Once stable swirls are built, they exhibit a mean velocity of 34 nm/s and a turnover time of short fragments of 11.5 s. From the velocity and the turnover time, the average length of the protofilaments can be estimated to 390 nm. However, ring formation is only observed at intermediate protein density regimes. At high protein density, a parallel network of filaments (nematic phase) is observed. (B) To guarantee chirality, attachment needs to have a perpendicular component to both the ring curvature and filament polarity to have a preferential binding face. In this case, the mts interacts with the flat surface on opposite sides of the FtsZ filament, so curvature is also in the opposite direction. (C) We here suggest that an intrinsic helical FtsZ shape, characterized by a radius and a pitch, can alternatively explain previous FtsZ-induced inwards/outwards deformations in the following way: Due to the intrinsic pitch, the growing filament would either pull up (left) or push down (right) the surface. On the contrary, if the surface is not deformable (SLB), the filament would experience a strain, get destabilized, and eventually break upon growth. GDP, guanosine diphosphate; GTP, guanosine triphosphate; GUV, giant unilamellar vesicles; mts, membrane-targeting sequence; SLB, supported lipid bilayer; YFP, yellow fluorescent protein.

We found that the most decisive factor for the emergence of dynamic vortices of FtsZ on membranes is the overall surface coverage by protein monomers and filaments (Fig 6A), which varies over time upon protein adsorption to the membrane (Fig 2) and is controlled by free Mg^2+^ concentration (Fig 3). Dynamic vortices appear primarily in an intermediate density regime (surface mean intensity: 450–1,000 arbitrary units [A.U.]) and isotropic bundles in a high-density regime (surface mean intensity > 1,000 A.U.). Transitions from highly dynamic vortices to isotropic bundles occur upon an increase in lateral contacts that arrest treadmilling filaments, increasing their mean effective length (as in the case of ZipA [15]). This was clearly observed in our single molecule assay (Fig 5B) that shows a slower turnover in the situation of dense isotropic bundles, i.e., longer filaments. Along these lines, the increase in lateral interactions at high free Mg^2+^ also explains the rapid formation of filaments at 5 mM free Mg^2+^ (Fig 3), since larger FtsZ assemblies bind to the membrane and interact with each other more frequently (Fig 5B). Presumably, the main reason why Loose and Mitchison failed to observe dynamic vortices in the case of the FtsZ-YFP-mts [15] is because of the high protein concentration used in their experiments (1.5 uM).

Our results are compatible with previous atomic force microscopy analysis of static structures formed by FtsZ polymers on mica as a function of protein concentration at the surface [23]. The protein concentration–dependent formation of dynamic FtsZ patterns also nicely correlates with a recent theoretical study suggesting that protein density at the membrane controls the formation of vortex patterns on membranes in a phase-like behavior. According to this, independent curved polar filaments showing chiral motion and repulsion can self-assemble into vortex or ringlike structures in an intermediate density regime. While at low protein densities filaments travel independently, at the high-density regime they form isotropic networks and jammed bundles [24].

By gradually increasing protein concentration on the membrane, we were able to investigate the initial formation phase of dynamic rings. At low density (surface mean intensity < 450 A.U.), curved and polar filaments initially emerge from nucleation points, which presumably are small attached filaments above a critical length. Intriguingly, the overall adsorption rates to the membrane at this stage are similar for protein concentrations of 0.2 μM and 0.5 μM (Fig 2A). Upon sufficiently high membrane coverage of nucleating filaments after the initial phase, protein binding from solution begins to scale with total (i.e., bulk) protein concentration. Whether nucleators are directly formed on the membrane after GTP addition or whether short polymers are formed in solution and an increase in affinity with growth brings them to the membrane cannot be determined based on our data and will be the topic of further investigation.

We found that filaments growing from nucleators are prone to fragmentation, resulting in free fragments that may stay connected to the membrane. There, they assemble with other attached filaments by diffusion and directional treadmilling, which ultimately results in closed rings in which the treadmilling continues. Our experiments demonstrate that treadmilling, particularly via destabilization of the trailing edge, is highly regulated by GTPase activity. When GTPase activity is switched off (FtsZ*[T108A]-YFP-mts), rings seemed to grow only from nucleation points and do not treadmill, at least on time scales found for FtsZ-YFP-mts (Fig 4C). Moreover, the residence time of single FtsZ*[T108A]-YFP-mts subunits in the filaments is comparable with the photobleaching control, implying that protein turnover is almost nonexistent (S6 Fig). It has been suggested that a kinetic and structural polarity at monomeric level and a GTP/GDP gradient are requirements for robust treadmilling [25]. As seen from our experiments of the initial vortex growth phase, a GTP/GDP gradient along the treadmilling direction is likely to result from the preferential addition of GTP subunits to the existing filaments at the polar front and a more likely GTP turnover toward the “older” tail.

In light of the role of GTPase activity for the formation of dynamic vortices, the measured velocity of the FtsZ + FtsA vortices reported by Loose and Mitchison is about 3-fold faster (108 nm/s) compared to our FtsZ-YFP-mts rings (34 nm/s). In addition, these authors reported a higher GTPase activity of the FtsZ + FtsA compared to the FtsZ-YFP-mts [15]. Nonetheless, it is not clear how variables such as GTPase activity and attachment strength influence the speed of rotation. For instance, our mutant mts-H-FtsZ-YFP has shown a considerable decrease in rotation speed compared to the FtsZ-YFP-mts. Remarkably, to observe mts-H-FtsZ-YFP dynamic rings, we had to increase the bulk concentration to 1.25 uM. This may be due to a reduced affinity for membranes, affecting the overall dynamics.

The most remarkable outcome of this study is the clear dependence of vortex chirality on the positioning of the membrane anchor, which, in turn, has severe effects on the topology of membrane deformation by FtsZ. Chirality is inverted by switching the membrane anchor from the C-terminus (clockwise) to the N-terminus (counterclockwise). Intriguingly, these two different mutants cause concave (C-terminal) or convex (N-terminal) deformations when bound to deformable liposomes [14]. To explain these different deformations, Erickson and colleagues have previously depicted FtsZ filaments as arc segments with a direction of membrane attachment either parallel or antiparallel to the vector of curvature. In order to support the here-observed chiral treadmilling of curved rings on planar membranes, however, attachment through the preferential binding face of the filaments needs to have a perpendicular component to both the ring curvature and filament polarity. Fig 6B shows a curved filament with a C-terminal (clear gray) and N-terminal (dark gray) face perpendicular to the curvature of the filament. Note that the mts is represented here with one parallel component to the curvature, as suggested by Erickson, and one perpendicular component to accommodate flat membrane binding. In this flat representation of a curved FtsZ filament, treadmilling is explained by a polar growth at the leading edge and a destabilization mechanism, driven by GTP hydrolysis, toward the GDP-enriched region at the trailing edge [25].

Nevertheless, due to the fact that curved structures can be either attached along their axis of apparent curvature (in vivo, Osawa and Erickson [14]) or perpendicular to their axis of primary curvature (as reported here), we have to conclude that either the membrane attachment of the filament is immensely flexible or—and this is more likely, based on previous structural investigations—that the filament does not have a single but rather more than one direction of curvature, like a helix or a twisted arc [19,26]. Indeed, a very similar geometry with more than one curvature direction has recently been reported for endosomal sorting complex required for transport III (ESCRTIII) filaments (nicely reviewed by Chiaruttini and Roux [27]). This is particularly intriguing as, among many other roles in eukaryotic and prokaryotic cells connected with membrane abscission, ESCRT is the alternative system to FtsZ with respect to cell division in Archaea [28,29].

Therefore, in light of evidence showing that FtsZ forms helical structures *in vivo* [30–33] and *in vitro* [19], we here suggest an alternative structural model to the one depicted in 6B (Fig 6C). We propose that an FtsZ filament with more than one main direction of curvature, such as a helix, would much more elegantly accommodate the combination of inward/outward deformations and chiral treadmilling for the opposite mts mutants. Such a corkscrew-like FtsZ filament can be simply described by an intrinsic radius and a pitch, in which the latter would reflect on the attachment direction (Fig 6C). On a deformable surface (deflated liposome), the growing filament would either pull up (left) or push down (right) the surface due to the respective pitch. The interplay between the elastic response of the membrane (increased membrane tension) and local changes in the helix radius due to GTP hydrolysis [34,35] could explain the stabilization of higher curvature or smaller radii regions (Fig 6C). On a nondeformable surface (SLB), however, since the surface is not resilient, the filament would experience a strain, get destabilized, and eventually break upon growth.

Thus, together with the very recent studies showing the linkage between treadmilling of FtsZ polymers and peptidoglycan synthesis in *E*. *coli* [4] and *B*. *subtilis* [5] cells, our findings shed new light on the interplay between FtsZ structure and treadmilling dynamics but may also hint to a direct mechanical link of these to bacterial division. The minimal system we used unambiguously shows that the observed chiral vortices are the result of intrinsic GTP-linked FtsZ polymerization dynamics on the membrane without the need of additional complex interactions with FtsA and ATP, pointing to a fascinating archetypal feature of this important structural protein. The reduced number of components allowed us to selectively determine the influence of key factors—e.g., the surface density of FtsZ—on the self-organization behavior, thus contributing to a much better mechanistic understanding of FtsZ’s dynamic architecture and its potential physiological implications.

## Materials and methods

### Mutants

Mutations in *ftsZ-YFP-mts* were constructed using site-directed mutagenesis. FW oligonucleotide was designed using the NEBaseChanger–Substitution (http://nebasechanger.neb.com/). RV oligonucleotide was obtained from the reverser complement sequence of the FW oligonucleotide. Oligonucleotides 5’- GGTGGTGGTGCCGGTACAGGT-3’ and 5’- ACCTGTACCGGCACCACCACC-3’ were used to generate FtsZ-YFP-mts-T108A mutant by replacing a Thr in position 108 by an Ala. Briefly, *ftsZ-YFP-mts* was first amplified using the FW and RV oligonucleotides in different PCR reactions, testing 3 different temperatures: 54°C, 58.5°C, and 65°C. In a second PCR reaction, the PCR products from the FW and RV oligonucleotides were mixed; also, 3 different temperatures were tested: 54°C, 58.5°C, and 65°C. After digestion with DpnI, the 3 PCR products were used to transform CH3-Blue competent cells. Five colonies were picked for DNA extraction and selected for sequencing.

### Protein purification

The FtsZ-YFP-mts, FtsZ*[T108A]-YFP-mts, and mts-H-FtsZ-YFP were purified as previously described [36]. Briefly, the protein was expressed from a pET-11b expression vector and transformed into *E*. *coli* strain BL21. ON overexpression was performed at 20°C for the proteins FtsZ-YFP-mts and FtsZ*[T108A]-YFP-mts and at 37°C for protein mts-H-FtsZ-YFP. Cells were lysed by sonication and separated by centrifugation. Then, protein was precipitated from the supernatant, adding 30% ammonium sulphate and incubating the mixture for 20 min on ice (slow shaking). After centrifugation and resuspension of the pellet, the protein was purified by anion exchange chromatography using a 5× 5 ml Hi-Trap Q-Sepharose column (GE Healthcare, 17515601). Purity of the protein was confirmed by SDS-PAGE and mass spectrometry.

### Small unilamellar vesicles (SUVs)

*E*. *coli* polar lipid extract (Avanti, AL, United States), initially dissolved in chloroform, was desiccated under a gas nitrogen stream. Chloroform traces were further removed by 1-h vacuum. This lipid film was hydrated with SLB-buffer (50 mM Tris-HCl, 150 mM KCl, pH 7.5) to reach a lipid concentration of 4 mg ml^−1^. After 10 min sonication, SUVs were obtained.

### SLBs

Glass coverslips #1.5 (Menzel, Germany) were cleaned in air plasma. Then, a plastic chamber was attached on a cleaned glass coverslip using ultraviolet-curable glue (Norland Optical Adhesive 63). SLBs were obtained by the method of vesicle fusion from SUVs on a glass surface as described elsewhere [37]. The SUV dispersion was diluted in SLB buffer (50 mM Tris-HCl at pH 7.5, 150 mM KCl) to 0.5 mg ml^−1^, of which 75μl was added to the reaction chamber. Adding CaCl_2_ to a final concentration of 3 mM promoted vesicle fusion and the formation of a lipid bilayer on the glass. The samples were incubated at 38°C for 20 min and then washed with prewarmed SLB buffer to remove nonfused vesicles. Lastly, a washing step with the reaction buffer (50 mM Tris-HCl at pH 7.5, 150 mM KCl and MgCl_2_ [5 mM or 1 mM]) was carried out in the sample.

### Self-organization assays

FtsZ-YFP-mts or FtsZ-YFP-mts mutants were added to the reaction buffer above the supported lipid membrane in the chamber. The final volume of the samples was approximately 200μl. FtsZ-YFP-mts was added to a final concentration of 0.5 μM or 0.2 μM. Polymerization was induced by adding GTP to a final concentration between 0.04 and 4 mM, as indicated in the text.

### TIRFM imaging

All experiments were performed on a WF1 GE DeltaVision Elite TIRFM (GE Healthcare Life Sciences, Germany) equipped with an OLYMPUS 100× TIRF objective (NA 1.49). The UltimateFocus feature of DeltaVision Elite maintains the focus plane constant in time. FtsZ-YFP-mts was excited with a 488 nm diode laser (10 mW, before objective) Fluorescence imaging is performed using a standard FITC filter set. Images were acquired with a PCO sCMOS 5.5 camera (PCO, Germany) controlled by the softWoRx Software (GE Healthcare Life Sciences, Germany). For time-lapse experiments, images were acquired every 3 or 10 s, with a 0.05 s exposure time, with light illumination shuttered between acquisitions.

### Image analysis and processing

Image analysis was carried out in MATLAB 2015 (MATLAB and Image Processing and Computer Vision Toolbox Release 2015a, The MathWorks, Inc., Natick, Massachusetts, USA) and processing with Fiji/ImageJ (Rasband, W. S., ImageJ, US National Institutes of Health, Bethesda, http://rsb.info.nih.gov/ij/, 1997–2007). Images correspond to an average of 5–10 frames from a time-series experiment. For the kymograph analysis, time-series acquisitions were filtered using a standard mean filter and were drift corrected (Image J). A MATLAB script allows the user to define a ring by providing 2 coordinates. Every ring is automatically fitted to a circle with radius r. Then, 3 trajectories corresponding to 3 concentric circles having radii r, r+1, and r−1 pixels are determined. At this point, the script will read the time-series data and calculate a kymograph for each time point and trajectory. The final kymograph corresponds to the average of the 3 different trajectories. To automatically calculate the slope, we first smooth the kymograph with a Savitzky-Golay filter of order 2 and enhance its contrast using a contrast-limited-adaptive-histogram-equalization (CLAHE) routine (MATLAB). Next, using Fourier analysis, we find the characteristic frequency for the patterns on the kymograph (S4 Fig). Finally, the slope corresponds to the change in phase at this frequency. Quality criteria are properly chosen to reject low-quality regions over the kymograph. To synchronize time-lapse acquisitions, the initial frame (time 0) was defined when surface mean intensity was around 200 A.U.

### Single-molecule imaging and residence time measurement

FtsZ-YFP-mts previously incubated 1:1 with the nanobody GFP-Booster Atto647N (ChromoTek, Germany) for at least 1 h at 4°C under agitation. To filter out nonbound nanobody, we centrifuge our protein in a 30 KDa Amicon unit. The GFP-Booster Atto647N was excited with a 640 nm diode laser (30 mW, before objective). Single molecule imaging was performed using a standard Cy5 filter set. After 10 min of GTP addition, a significant number of spots in the single molecule channel (Atto647N) were observed and imaged at a rate of 1 fps or 3 fps with 0.3 s exposure time. To improve imaging conditions, we added 10 nM protocatechuate-dioxygenase (PCD) and 2 mM 3,4-protocatechuicacid (PCA) as an oxygen-scavenging system. To determine the position of every single molecule and calculate its residence time, we employed a MATLAB routine designed by Weimann and Ganzinger [38]. Briefly, a bandpass filter was used to remove low- and high-frequency noise. Then, single molecule positions with intensity above a user-defined threshold were determined by their brightness-weighted centroid. The detection algorithm is highly efficient for detecting particles with a signal-to-noise ratio above 1.5. The user-defined threshold was chosen to detect the largest number of spots and kept constant for all experiments. Single molecules were tracked among consecutive frames in an area given by a radius of 10 pixels (pixel size = 0.042 μm). Thus, the residence time is defined as the time that the particle stays in this area before its signal vanishes.

To calculate the mean residence time, we calculated the probability as a function of time *t* to obtain a loss of signal event at times ≤ *t* (cumulative probability, S6A Fig). We fitted to a double exponential function $Ae^{-kt}\;+\;Be^{-k_{p}t}$, in which k refers to the inverse of the mean residence time, and $k_{p}$ corresponds to the photobleaching rate. A and B are constrained, since the photobleaching contribution is limited to be between 0.2–0.25 in the fitting routine for all conditions (MATLAB). The photobleaching rate was calculated as $k_{p}\;=\;0.031s^{-1}$ using a single exponential fit shown in S6B Fig.

Events shorter than 2 frames are below the accuracy of our method and were not included in the statistics. The cumulative probability was measured for 5 different experiments having a total number of events (*N*) in each experiment. For GDP forms: 5 mM Mg^2+^, *N* varies in the range of 3,000–5,300 events (1 fps) and 660–8,800 events (3 fps); 1 mM Mg^2+^, *N* = 3,000–11,000 (3 fps). For GTP forms: 0.04 mM GTP, *N* = 1,300–3,000 (1 fps); and 4 mM GTP, *N* = 1,200–6,700. In the case of FtsZ*[T108A]-YFP-mts at 4 mM GTP, *N* = 180–800.

## Supporting information

S1 Fig FtsZ-YFP-mts sedimentation velocity analysis. c(S) sedimentation coefficient distributions obtained for the chimeric FtsZ variant (7 μM) obtained from experiments done in working buffer at the following conditions: 4 mM GTP and 5 mM Mg^2+^ (A), 4 mM GTP but no Mg^2+^ added (B), 0.05 mM GDP and 5 mM Mg^2+^ (C), and 0.05 mM GDP and no Mg^2+^ added (D). These experiments show that FtsZ-YFP-mts is a well-behaved self-associating protein. In the absence of GTP and/or Mg^2+^ (B–D), the protein exists mainly as a slowly sedimenting species with s-value of around 6S (C–D), compatible with the heterodimeric FtsZ-YFP form. In the presence of both GTP and Mg^2+^ (A), most of the protein (70%) sediments as a polydisperse mixture of higher-order species with an average s-value of 20 +/− 5 S. These results are important because they allowed us to control the association state of the membrane-targeted FtsZ variant by GTP and Mg^2+^, which was crucial to obtain the reproducible dynamic ringlike structures on the bilayers shown in this work (see main text). The broad distribution of higher-order species shown in (A) contrast with the sharp s-values observed in previous studies from the Rivas lab under specific experimental conditions of protein and buffer composition [17]. These differences in sedimentation coefficient distributions of FtsZ under assembly-promoting conditions could, in part, be related to the presence of the mts-tag. However, they are also compatible with the behavior of self-assembling systems as plastic as FtsZ, in which it would take only a very small free energy perturbation to produce large changes in the relative abundance of the species present as higher-order oligomers [17]. GDP, guanosine diphosphate; GTP, guanosine triphosphate; mts, membrane-targeting sequence; YFP, yellow fluorescent protein.
(TIF)

S2 Fig Representative images of FtsZ-YFP-mts at low (left panel) and high (right panel) protein concentrations. Only short filaments could be detected at 0.1 μM; no further structures were later observed. On the contrary, when 1 μM of FtsZ-TFP-mts is added, polymer networks were observed almost instantly at the vicinity of the membrane. Dynamic rings were only noticed at intermediated protein concentrations. mts, membrane-targeting sequence; TFP, teal fluorescent protein; YFP, yellow fluorescent protein.
(TIF)

S3 Fig Effect of GTP concentration on the FtsZ-YFP-mts rings formation. (A) Images of 0.2 μM FtsZ-YFP-mts bundles after the addition of 0.4 mM GTP and 0.04 mM GTP showing long filaments with a parallel arrangement. Images of upper panels were taken 1–2 min (initial acquisition) after GTP addition. Lower panels represent images after 10 min. (B) Bar plot of the mean fluorescence intensity of the different GTP concentrations at initial time of triggering and 10 min later. GTP, guanosine triphosphate; mts, membrane-targeting sequence; YFP, yellow fluorescent protein.
(TIF)

S4 Fig Fourier analysis allows automatic calculation of the slope of the kymograph. (A) A representative kymograph after a Savitzky-Golay filter and enhance contrast using a CLAHE routine. To discard low-quality regions, the analysis is made over 50 vertical pixels (marked region in A). (B) The FFT shows a clear peak at approximately 0.02 Hz. (C) Then, to calculate the slope, we measure the change in phase by linear fit. Regions are rejected when (a) the peak in the FFT spectrum is lower than 10-fold the mean of the FFT data or (b) linear fit with R^2^ < 0.95. CLAHE, contrast-limited-adaptive-histogram-equalization; FFT, Fourier transformation spectrum.
(TIF)

S5 Fig GTPase activity of FtsZ-YFP-mts (5 μM) or FtsZ*[T108A]-YFP-mts (5 μM) in the absence or presence of phospholipids (4 mg/ml). The corresponding rates were normalized to the GTP activity of FtsZ-YFP-mts in the absence of phospholipids. We observed that the GTPase activity of FtsZ*[T108A]-YFP-mts was almost zero. GTPase activities were determined using the BIOMOL GRENN assay (Enzo). Error bars correspond to standard deviation from 3 different experiments. GTP, guanosine triphosphate; mts, membrane-targeting sequence; YFP, yellow fluorescent protein.
(TIF)

S6 Fig (A) Cumulative probability of the residence time distributions for (a) 4 mM GTP 1 mM free Mg^2+^ and (b) 0.04 mM GTP 1 mM free Mg^2+^ having an acquisition rate (1 fps). Inset: Cumulative residence time distribution for GDP forms with a faster acquisition rate (3 fps) at (c) 1 mM free Mg^2+^ and (d) 5 mM free Mg^2+^. Note that the residence time distributions for GDP at 5 mM Mg^2+^ (blue circles) are equivalent at 1 fps and 3 fps. (e) represents photobleaching decay. Curves were fitted to a double exponential function to calculate the mean residence time having a constant photobleaching contribution. Further details are under “Materials and methods.” (B) Cumulative probability of the residence time distribution for FtsZ*[T108A]-YFP-mts (closed squares). In the same plot, the photobleaching timescale of fixed nanobodies is shown (empty inverted triangles). fps, frames per second; GDP, guanosine diphosphate; GTP, guanosine triphosphate; mts, membrane-targeting sequence; YFP, yellow fluorescent protein.
(TIF)

S1 Movie FtsZ-YFP-mts assembles into circular vortices as a time-dependent process at a protein concentration of 0.5 μM. After GTP addition, monomers are released into solution in which the polymerization is triggered. Then, short protofilaments from solution slowly return to the membrane, forming small structures with high lateral diffusion (5 min). After 10 min, those short filaments formed unstable and small circular structures that transiently open and close. At later times, ringlike structures with a defined center become predominant (15 min). In long-time regime, rings become larger and thicker. (Images acquired every 10 s and displayed at 15 fps). fps, frames per second; GTP, guanosine triphosphate; mts, membrane-targeting sequence; YFP, yellow fluorescent protein.
(MP4)

S2 Movie FtsZ-YFP-mts shows a polar clockwise growth from a nucleation point. Right panel corresponds to the square area 1 showed in left panel. mts, membrane-targeting sequence; YFP, yellow fluorescent protein.
(MP4)

S3 Movie FtsZ-YFP-mts forms filaments with a specific direction that can glide by treadmilling. mts, membrane-targeting sequence; YFP, yellow fluorescent protein.
(MP4)

S4 Movie Left panel: Single rings of FtsZ-YFP-mts behave as rotating vortices, showing a chiral clockwise rotation as a result of treadmilling at a protein concentration of 0.5 μM. Middle panel: FtsZ chimera with no GTPase activity (FtsZ*[T108A]-YFP-mts) forms defined rings without apparent rotation at a protein concentration of 0.2 μM. This FtsZ chimera shows rings with similar size to the ones found with FtsZ-YFP-mts. Right panel: The protein chimera mts-H-FtsZ-YFP, which has the mts in the N-terminus, creates dynamic rings that strikingly appeared to rotate in the counterclockwise direction at a protein concentration of 1.25 μM. (Images acquired every 3 s and displayed at 16 fps). fps, frames per second; GTP, guanosine triphosphate; mts, membrane-targeting sequence; YFP, yellow fluorescent protein.
(MP4)

S5 Movie FtsZ filaments growth from nucleation points with different dynamics. Left panel corresponds to FtsZ-YFP-mts; right panel corresponds to FtsZ*[T108A]-YFP-mts. Both acquisitions have been started right after GTP addition. GTP, guanosine triphosphate; mts, membrane-targeting sequence; YFP, yellow fluorescent protein.
(MP4)

S1 Text Contains the supplementary methods of this work. (PDF)

S1 Data Contains raw numerical values that underlie the summary data displayed in the following figure panels: Figs 2B, 3, 4D, 5B and S3B, S5, and S6. (XLSX)

## Abbreviations

A.U.: arbitrary units
CLAHE: contrast-limited-adaptive-histogram-equalization
ESCRTIII: endosomal sorting complex required for transport III
FRAP: fluorescence recovery after photobleaching
GDP: guanosine diphosphate
GFP: green fluorescent protein
GTP: guanosine triphosphate
GUV: giant unilamellar vesicle
mts: membrane-targeting sequence
PCA: protocatechuicacid
PCD: protocatechuate-dioxygenase
SLB: supported lipid bilayer
SUV: small unilamellar vesicle
TIRFM: total internal reflection fluorescence microscopy
YFP: yellow fluorescent protein
