# Supplementary figures and images for: Treadmilling analysis reveals new insights into dynamic FtsZ ring architecture
Source: PLoS Biol. 2018 May 18;16(5):e2004845. doi: 10.1371/journal.pbio.2004845 (PMC5979038; doi:10.1371/journal.pbio.2004845)

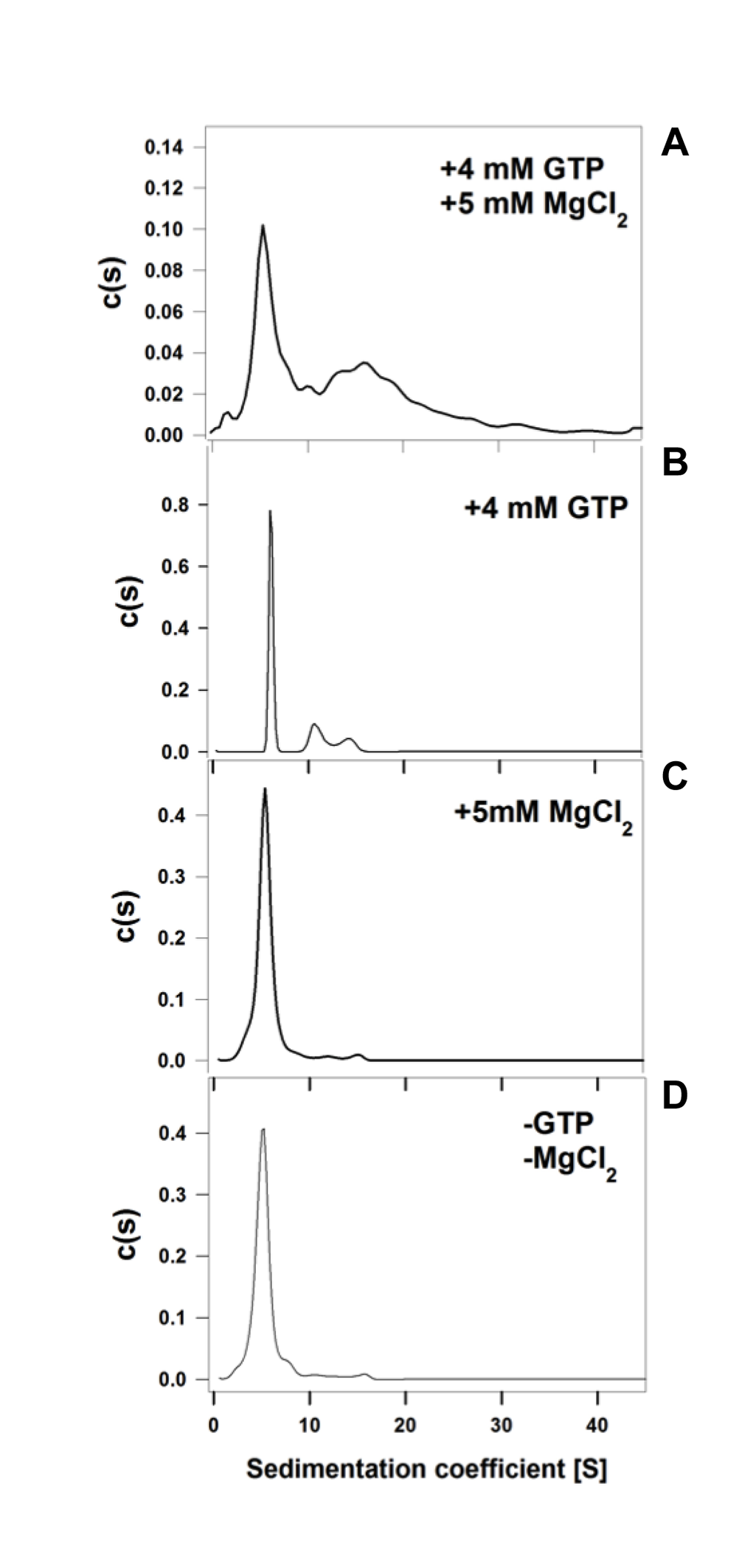

Supplement: S1 Fig — c(S) sedimentation coefficient distributions obtained for the chimeric FtsZ variant (7 μM) obtained from experiments done in working buffer at the following conditions: 4 mM GTP and 5 mM Mg2+ (A), 4 mM GTP but no Mg2+ added (B), 0.05 mM GDP and 5 mM Mg2+ (C), and 0.05 mM GDP and no Mg2+ added (D). These experiments show that FtsZ-YFP-mts is a well-behaved self-associating protein. In the absence of GTP and/or Mg2+ (B–D), the protein exists mainly as a slowly sedimenting species with s-value of around 6S (C–D), compatible with the heterodimeric FtsZ-YFP form. In the presence of both GTP and Mg2+ (A), most of the protein (70%) sediments as a polydisperse mixture of higher-order species with an average s-value of 20 +/− 5 S. These results are important because they allowed us to control the association state of the membrane-targeted FtsZ variant by GTP and Mg2+, which was crucial to obtain the reproducible dynamic ringlike structures on the bilayers shown in this work (see main text). The broad distribution of higher-order species shown in (A) contrast with the sharp s-values observed in previous studies from the Rivas lab under specific experimental conditions of protein and buffer composition [17]. These differences in sedimentation coefficient distributions of FtsZ under assembly-promoting conditions could, in part, be related to the presence of the mts-tag. However, they are also compatible with the behavior of self-assembling systems as plastic as FtsZ, in which it would take only a very small free energy perturbation to produce large changes in the relative abundance of the species present as higher-order oligomers [17]. GDP, guanosine diphosphate; GTP, guanosine triphosphate; mts, membrane-targeting sequence; YFP, yellow fluorescent protein. (TIF) [file pbio.2004845.s001.tif]

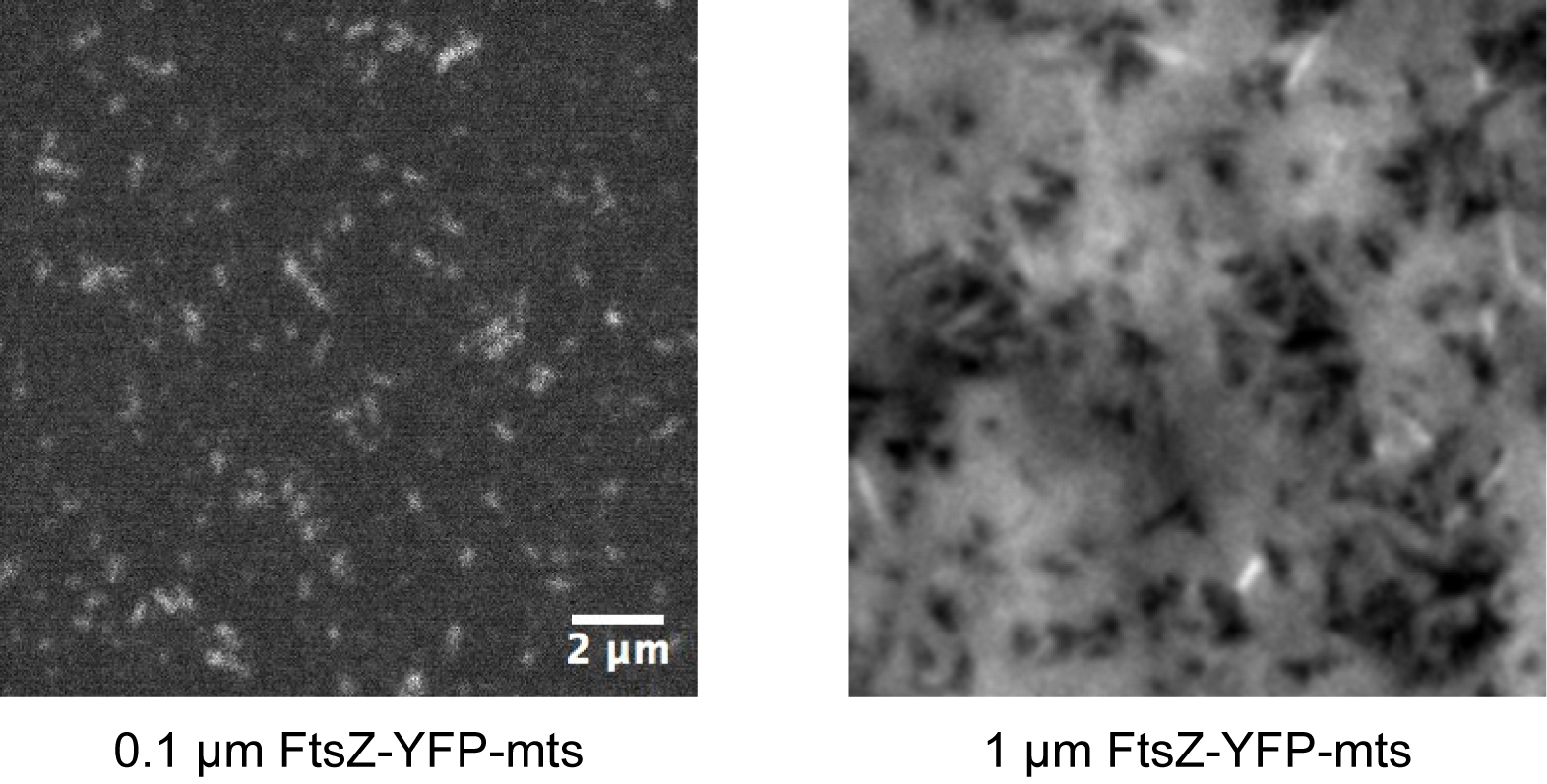

Supplement: S2 Fig — Only short filaments could be detected at 0.1 μM; no further structures were later observed. On the contrary, when 1 μM of FtsZ-TFP-mts is added, polymer networks were observed almost instantly at the vicinity of the membrane. Dynamic rings were only noticed at intermediated protein concentrations. mts, membrane-targeting sequence; TFP, teal fluorescent protein; YFP, yellow fluorescent protein. (TIF) [file pbio.2004845.s002.tif]

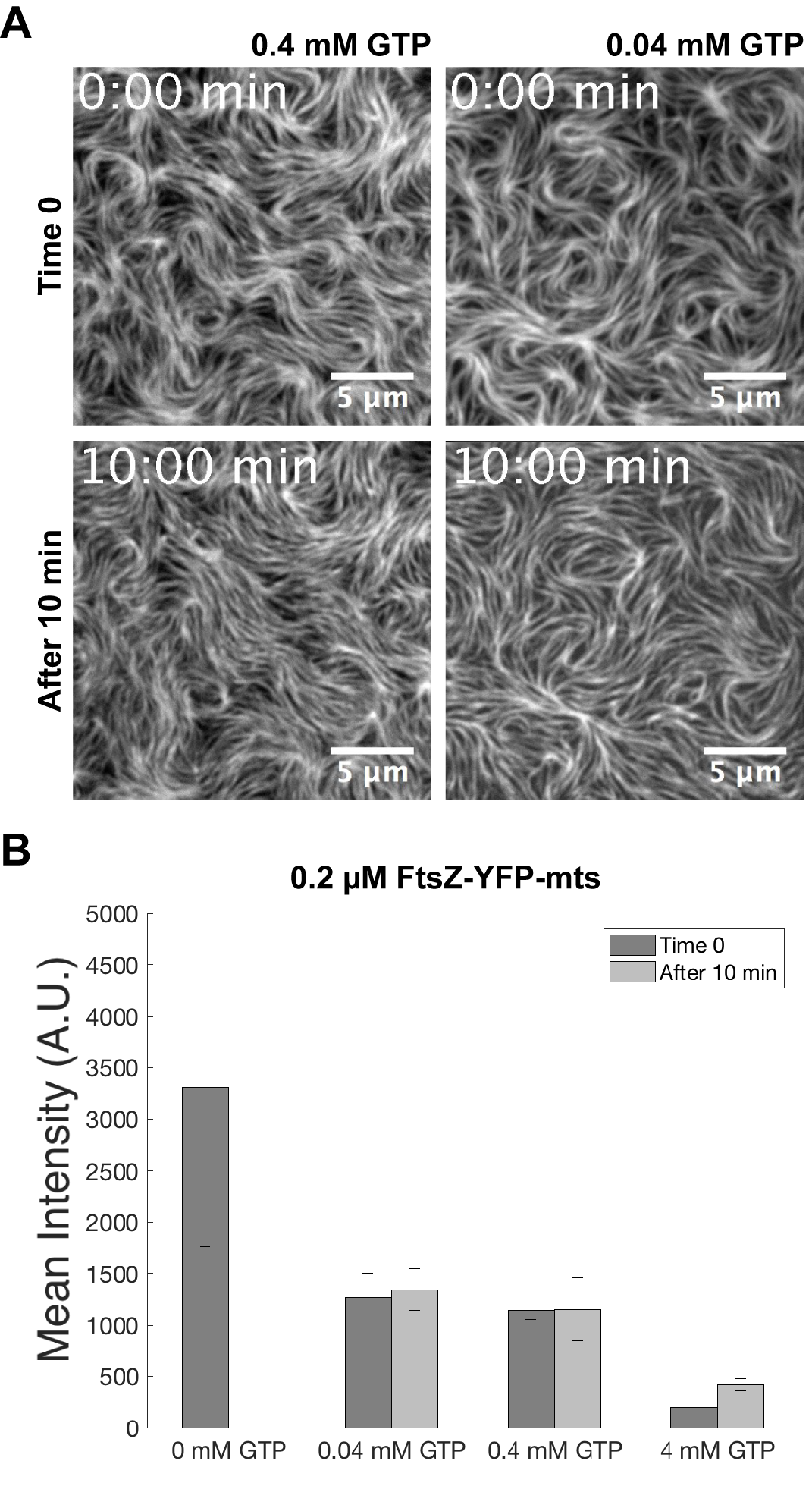

Supplement: S3 Fig — (A) Images of 0.2 μM FtsZ-YFP-mts bundles after the addition of 0.4 mM GTP and 0.04 mM GTP showing long filaments with a parallel arrangement. Images of upper panels were taken 1–2 min (initial acquisition) after GTP addition. Lower panels represent images after 10 min. (B) Bar plot of the mean fluorescence intensity of the different GTP concentrations at initial time of triggering and 10 min later. GTP, guanosine triphosphate; mts, membrane-targeting sequence; YFP, yellow fluorescent protein. (TIF) [file pbio.2004845.s003.tif]

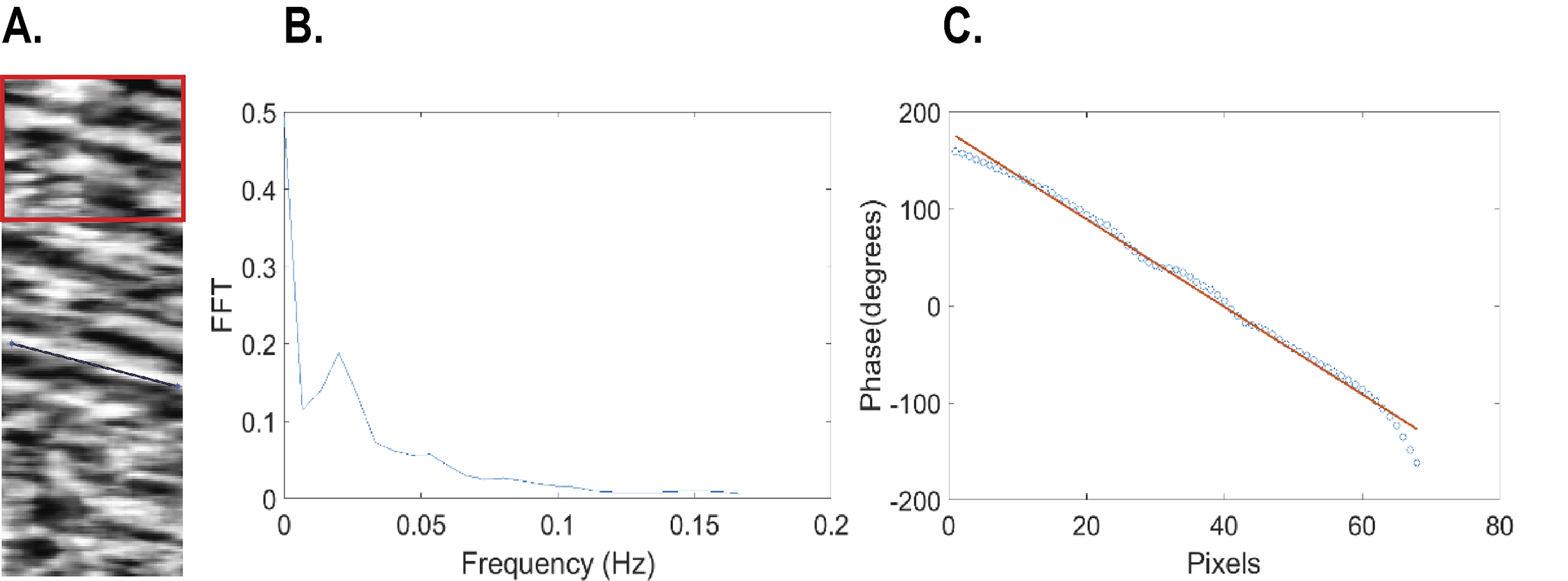

Supplement: S4 Fig — (A) A representative kymograph after a Savitzky-Golay filter and enhance contrast using a CLAHE routine. To discard low-quality regions, the analysis is made over 50 vertical pixels (marked region in A). (B) The FFT shows a clear peak at approximately 0.02 Hz. (C) Then, to calculate the slope, we measure the change in phase by linear fit. Regions are rejected when (a) the peak in the FFT spectrum is lower than 10-fold the mean of the FFT data or (b) linear fit with R2 < 0.95. CLAHE, contrast-limited-adaptive-histogram-equalization; FFT, Fourier transformation spectrum. (TIF) [file pbio.2004845.s004.tif]

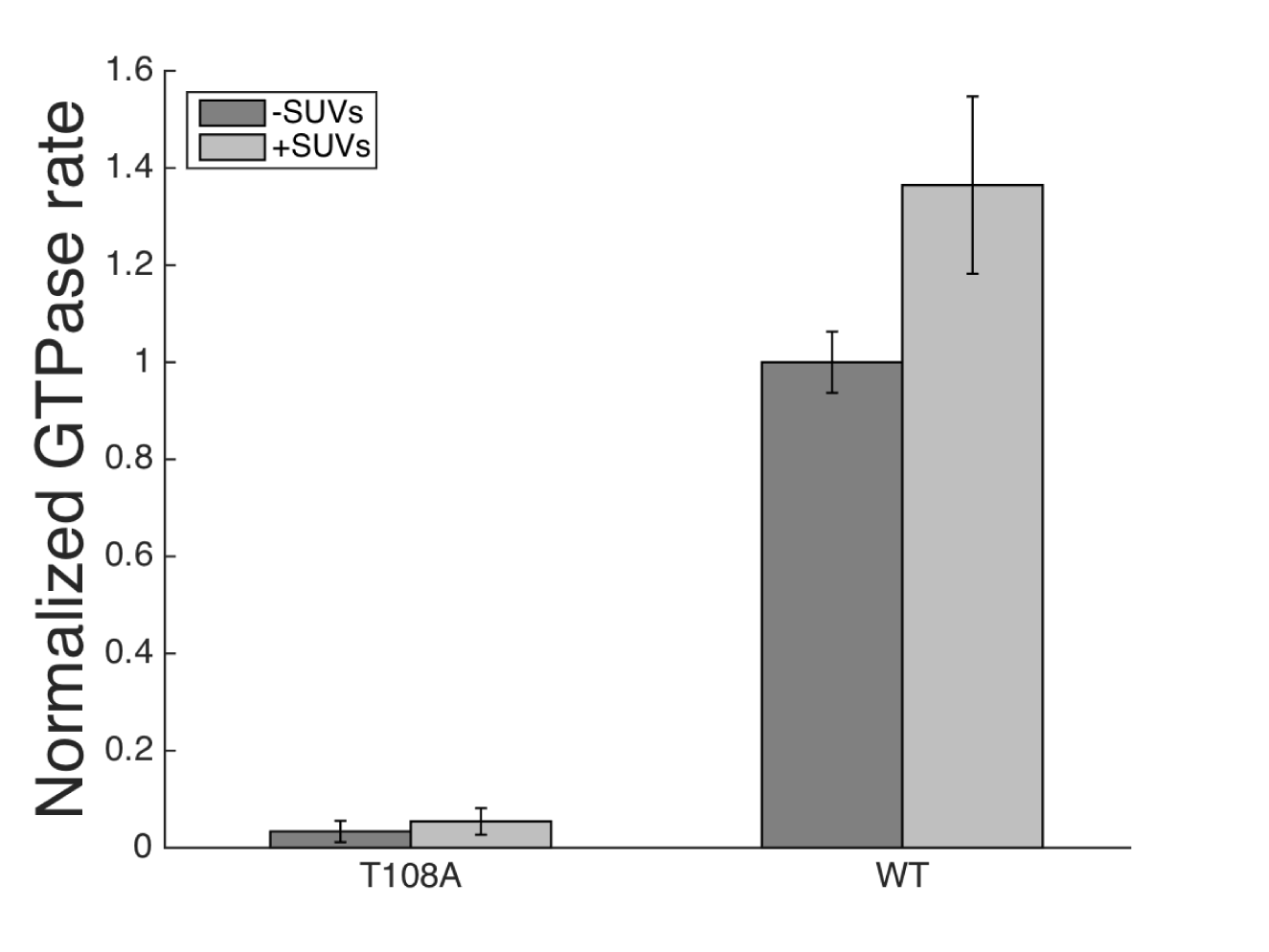

Supplement: S5 Fig — The corresponding rates were normalized to the GTP activity of FtsZ-YFP-mts in the absence of phospholipids. We observed that the GTPase activity of FtsZ*[T108A]-YFP-mts was almost zero. GTPase activities were determined using the BIOMOL GRENN assay (Enzo). Error bars correspond to standard deviation from 3 different experiments. GTP, guanosine triphosphate; mts, membrane-targeting sequence; YFP, yellow fluorescent protein. (TIF) [file pbio.2004845.s005.tif]

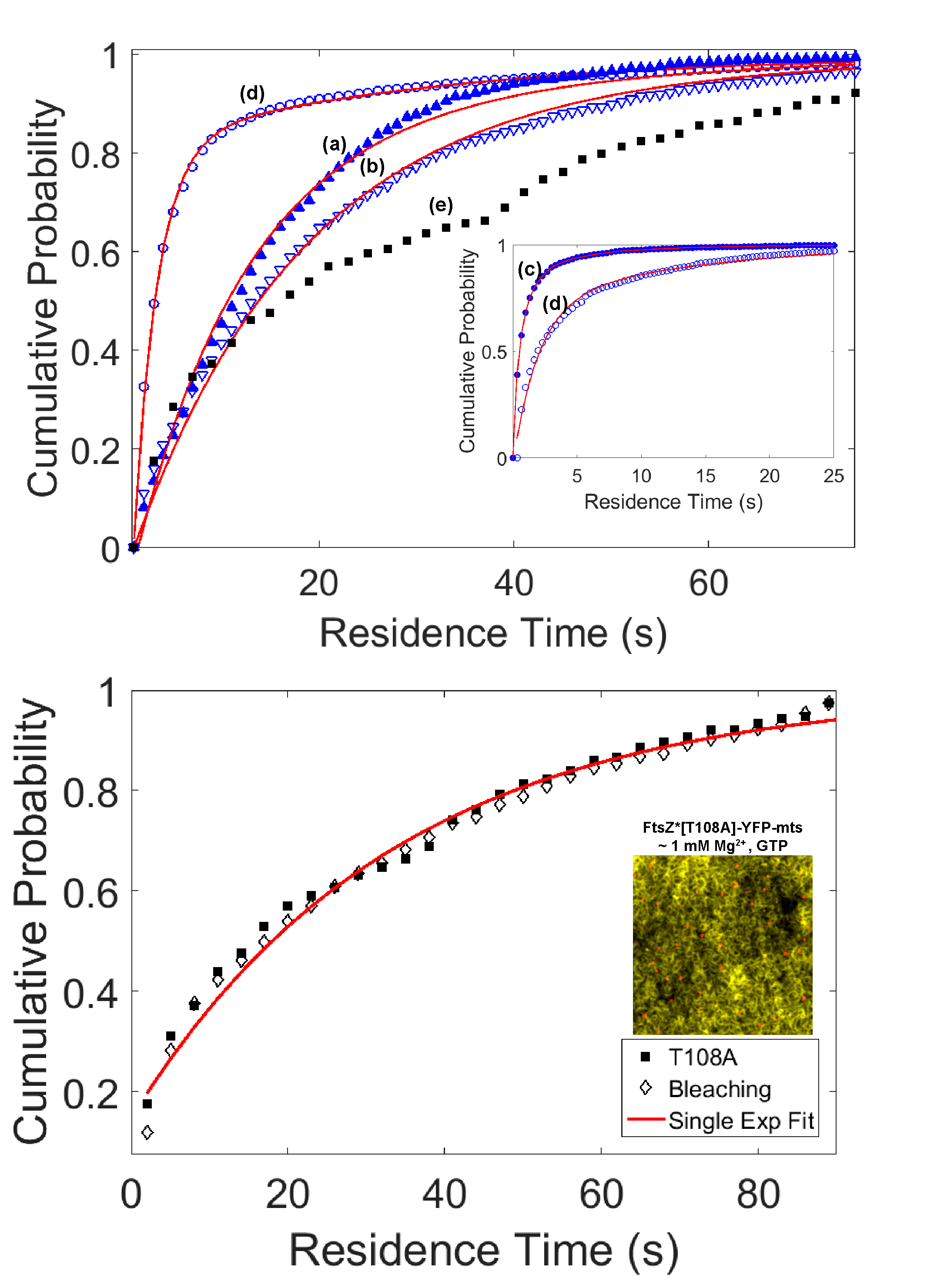

Supplement: S6 Fig — (A) Cumulative probability of the residence time distributions for (a) 4 mM GTP 1 mM free Mg2+ and (b) 0.04 mM GTP 1 mM free Mg2+ having an acquisition rate (1 fps). Inset: Cumulative residence time distribution for GDP forms with a faster acquisition rate (3 fps) at (c) 1 mM free Mg2+ and (d) 5 mM free Mg2+. Note that the residence time distributions for GDP at 5 mM Mg2+ (blue circles) are equivalent at 1 fps and 3 fps. (e) represents photobleaching decay. Curves were fitted to a double exponential function to calculate the mean residence time having a constant photobleaching contribution. Further details are under “Materials and methods.” (B) Cumulative probability of the residence time distribution for FtsZ*[T108A]-YFP-mts (closed squares). In the same plot, the photobleaching timescale of fixed nanobodies is shown (empty inverted triangles). fps, frames per second; GDP, guanosine diphosphate; GTP, guanosine triphosphate; mts, membrane-targeting sequence; YFP, yellow fluorescent protein. (TIF) [file pbio.2004845.s006.tif]
