# Supplementary material for: Treadmilling analysis reveals new insights into dynamic FtsZ ring architecture
Source: PLoS Biol. 2018 May 18;16(5):e2004845. doi: 10.1371/journal.pbio.2004845 (PMC5979038; doi:10.1371/journal.pbio.2004845)
Supplement: S1 Text — (PDF) [file pbio.2004845.s012.pdf]

SUPPLEMENTAY METHODS

Sedimentation velocity. Sedimentation velocity analysis of FtsZ-YFP-mts was performed at 0.5 g/L protein concentration in 50 mM Tris-HCl, 150 mM KCl, 5 mM $MgCl_2$, pH 7.5 buffer. The experiments were carried out at 38,000 rpm and at 20 ºC in an XL-I analytical ultracentrifuge (Beckman-Coulter Inc.) equipped with a UV-VIS detection system, an An-50 Ti rotor and 12 mm double-sector centerpieces. Sedimentation profiles were registered every 5 min. The sedimentation coefficient distributions were calculated by least-squares boundary modeling of sedimentation velocity data using the c(s) method as implemented in the SEDFIT program (Schuck, P., 2000). These S-values were corrected to standard conditions (water, 20 ºC and infinite dilution) to get the corresponding standard s 20,w using the software SEDNTERP (Holde, v.E.v.,1985). The frictional ratio, f/f0, was calculated for the monomer using this software.

GTPase activity. FtsZ GTPase activity was determined using the BIOMOL GREEN reagent for phosphate detection (Enzo). In brief FtsZ at 5 µM concentration is measured every 20 seconds after adding 1 mM GTP for a total of 7 time points. After 15 minutes of incubation with Biomol Green, the samples are measured OD_{620nm}. The data is later fit to a standard curve of 40 µM phosphate.

Schuck, P., Size-distribution analysis of macromolecules by sedimentation velocity ultracentrifugation and lamm equation modeling. Biophys J, 2000. 78(3): p. 1606-19.

Holde, v.E.v., Sedimentation. Physical biochemistry 2 edit.Prentice-Hall, Englewood Cliffs, NJ., 1985: p. 110-136.
